# Monitoring Radiofrequency Ablation Using Real-Time Ultrasound Nakagami Imaging Combined with Frequency and Temporal Compounding Techniques

**DOI:** 10.1371/journal.pone.0118030

**Published:** 2015-02-06

**Authors:** Zhuhuang Zhou, Shuicai Wu, Chiao-Yin Wang, Hsiang-Yang Ma, Chung-Chih Lin, Po-Hsiang Tsui

**Affiliations:** 1 College of Life Science and Bioengineering, Beijing University of Technology, Beijing, China; 2 Department of Medical Imaging and Radiological Sciences, College of Medicine, Chang Gung University, Taoyuan, Taiwan; 3 Graduate Institute of Clinical Medical Sciences, College of Medicine, Chang Gung University, Taoyuan, Taiwan; 4 Department of Computer Science and Information Engineering, Chang Gung University, Taoyuan, Taiwan; 5 Medical Imaging Research Center, Institute for Radiological Research, Chang Gung University and Chang Gung Memorial Hospital at Linkou, Taoyuan, Taiwan; University of Minnesota, UNITED STATES

## Abstract

Gas bubbles induced during the radiofrequency ablation (RFA) of tissues can affect the detection of ablation zones (necrosis zone or thermal lesion) during ultrasound elastography. To resolve this problem, our previous study proposed ultrasound Nakagami imaging for detecting thermal-induced bubble formation to evaluate ablation zones. To prepare for future applications, this study (i) created a novel algorithmic scheme based on the frequency and temporal compounding of Nakagami imaging for enhanced ablation zone visualization, (ii) integrated the proposed algorithm into a clinical scanner to develop a real-time Nakagami imaging system for monitoring RFA, and (iii) investigated the applicability of Nakagami imaging to various types of tissues. The performance of the real-time Nakagami imaging system in visualizing RFA-induced ablation zones was validated by measuring porcine liver (*n* = 18) and muscle tissues (*n* = 6). The experimental results showed that the proposed algorithm can operate on a standard clinical ultrasound scanner to monitor RFA in real time. The Nakagami imaging system effectively monitors RFA-induced ablation zones in liver tissues. However, because tissue properties differ, the system cannot visualize ablation zones in muscle fibers. In the future, real-time Nakagami imaging should be focused on the RFA of the liver and is suggested as an alternative monitoring tool when advanced elastography is unavailable or substantial bubbles exist in the ablation zone.

## Introduction

Hepatocellular carcinoma (HCC) is the most common type of liver cancer. Surgical resection and liver transplantation are the two primary methods for HCC treatment [1]. However, not every HCC patient is a suitable candidate for surgical treatment. Radiofrequency ablation (RFA), a minimally invasive procedure, has become the most frequently used alternative for treating unresectable liver tumors [2]. RFA involves inserting a needle-like radiofrequency (RF) electrode into a tumor to deliver a strong alternating electrical current, which agitates ions and increases the temperature to induce the coagulative necrosis of tissues surrounding the RF electrode. Image guidance when inserting the RF electrode into the target location of the tumor is indispensable for RFA treatment. Among the various medical imaging modalities, ultrasound B-mode imaging is widely used for guiding the insertion of the RF electrode because it can feedback the electrode location in real time [3].

RFA induce gas bubbles in ablation zones (necrosis zone or thermal lesion) because ablation heating raises the tissue temperature to near the boiling point [4], generating gas bubbles as strong acoustic scatterers that interact with incident ultrasound to produce hyperechoic regions in B-scans. Consequently, after using ultrasound to guide the insertion of the RF electrode, clinicians can employ the B-scan to observe the bubble-related hyperechoic region and preliminarily evaluate the ablation zones. However, the brightness of the B-mode image depends on the settings of the ultrasound imaging system (e.g., gain, time—gain compensation, and signal and image processing); thus, the performance of the B-scan in monitoring the RFA is operator-dependent. Moreover, tissue necrosis and bubble formation caused by RFA generates posterior acoustic shadowing in B-mode images, and thus, visualizing ablation zones is difficult, a topic that has been discussed in previous studies [4,5].

To complement B-mode images used in monitoring RFA, ultrasound elastography techniques have been extensively examined [6]. The fundamental principle of ultrasound elastography in monitoring RFA is that the ablated tissues are stiffer compared with normal, untreated tissues [6,7]. Ultrasound elastography approaches used for monitoring RFA can be classified into four types [6], namely quasistatic elastography [8–12], sonoelastography [13], acoustic radiation force elastography [14–17], and applicator motion elastography [18–23]. Ultrasound elastography has gradually become the predominant technique used for imaging RFA [8–23]. However, ultrasound elastography techniques require special architectures or complex instruments, a requirement that is considerable problems in numerous developing countries, particularly in regions with limited resources [24,25].

To promote imaging-aided diagnosis, developing a real-time functional imaging device on the basis of a conventional ultrasound imaging system (i.e., pulse—echo system) to noninvasively monitor RFA is crucial. In our previous study, we proposed a strategy that was based on ultrasound Nakagami imaging to characterize RFA-induced ablation zones [26]. The ultrasound Nakagami imaging technique has been applied to monitor ultrasound ablation (i.e., high-intensity focused ultrasound) [27–29]. Briefly, a Nakagami image is a parametric map consisting of a Nakagami parameter [30], which highly depends on the arrangements and concentrations of scatterers in a scattering medium. During RFA, the bubbles formed in an ablation zone through a thermal effect may be attributed to an increased concentration of scatterers, which further induces a change in the statistical distribution of backscattered signals and a corresponding increase in the Nakagami parameter [26]. In particular, the algorithm of Nakagami imaging is fully compatible with the standard pulse—echo ultrasound system.

To facilitate future clinical applications and promote image-aided monitoring, the following three critical problems must be resolved and clarified: (i) The data processing and ablation zone visualization performed in previous studies were offline [26–29]. The ultrasound Nakagami imaging algorithm must be integrated into a clinical scanner to test the computation and performance of Nakagami imaging in real-time monitoring. (ii) Gas bubble formation is constant during the RFA of a tissue. To improve the sensitivity of the conventional Nakagami image and collect sufficient information from bubbles, signal processing approaches and multiple Nakagami image frames obtained at various time points should be used together to construct the final image. Thus, an innovative algorithmic scheme for Nakagami imaging is necessary. (iii) The ability of ultrasound Nakagami imaging in visualizing ablation zones is attributed to RFA-induced bubbles that act as natural contrast agents, contributing strong backscattered echoes. Different types of tissues may produce varied bubble effects during RFA. In other words, the applicability of Nakagami imaging for various types of tissues should be investigated.

In this study, we developed a real-time ultrasound Nakagami imaging system for RFA monitoring. In particular, we designed a complete algorithmic scheme on the basis of the frequency and temporal compounding of multiple Nakagami images to enhance ablation zone visualization. Moreover, experiments on porcine liver and muscle tissues were conducted to examine the effects of tissue type on using Nakagami imaging to monitor RFA. The results were used to explain the physical mechanism of Nakagami imaging in visualizing RFA-induced ablation zones and to discuss the engineering novelties and scientific contributions of this study.

## Materials and Methods

### Nakagami model

In the Nakagami statistical model, the probability density function of ultrasonic backscattered envelope *R* is expressed by [30]

$$f(r)=\frac{2m^{m}r^{2m-1}}{\Gamma (m)\Omega^{m}}\exp\left(-\frac{m}{\Omega }r^{2}\right)U(r),$$ (1)

where Γ(∙) and U(∙) are the gamma function and unit step function, respectively. Let E(∙) denote the statistical mean. The scaling parameter Ω and Nakagami parameter *m* associated with the Nakagami distribution can be respectively obtained from

$$\Omega =E(R^{2})$$ (2)

and

$$m=\frac{{\left[E(R^{2})\right]}^{2}}{E{\left[R^{2}-E(R^{2})\right]}^{2}}.$$ (3)

**Fig 1:**
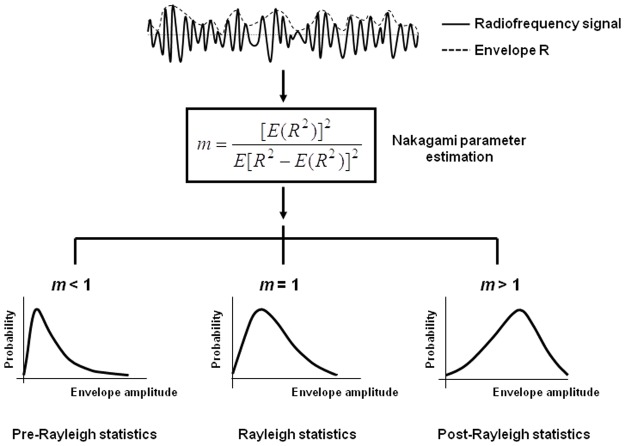
Raw ultrasound backscatter signals (i.e., radiofrequency signals) and the corresponding envelope signal used to estimate the Nakagami parameter *m* for modeling the statistical distribution of backscatter envelope *R*. Different Nakagami parameters indicate varying envelope statistics caused by various properties of scatterers in the tissue.

The Nakagami parameter *m*, estimated using backscattered envelopes, is a shape parameter used to describe the statistical distribution of the backscattered envelopes (Fig. 1). The variation of the Nakagami parameter from 0 to 1 indicates changes in the envelope statistics from pre-Rayleigh to Rayleigh distributions. The Nakagami parameters exceeding 1 indicate the backscattered statistics of post-Rayleigh distributions. The Nakagami distribution is a general model for ultrasonic backscattering [30].

### Conventional Nakagami imaging

Briefly, the Nakagami image is based on the Nakagami parameter map, which is constructed using a square sliding window to process the envelope image. This involves two principle steps: (i) A square window in the envelope image is used to collect the local backscattered envelopes and estimate the local Nakagami parameter, which is designated as the new pixel located in the center of the window. (ii) Let the window move throughout the entire envelope image in steps of a certain number of pixels corresponding to a window overlap ratio, and repeat Step 1 to construct the map of the local Nakagami parameter. A previous study suggested that the side length of the square window that can simultaneously satisfy the image resolution and stable parameter estimation is three times the pulse length of a transducer [31]. The previous study has validated the feasibility of adopting conventional Nakagami imaging to detect RFA-induced ablation zones [26].

### Frequency and temporal compounding Nakagami imaging

**Fig 2:**
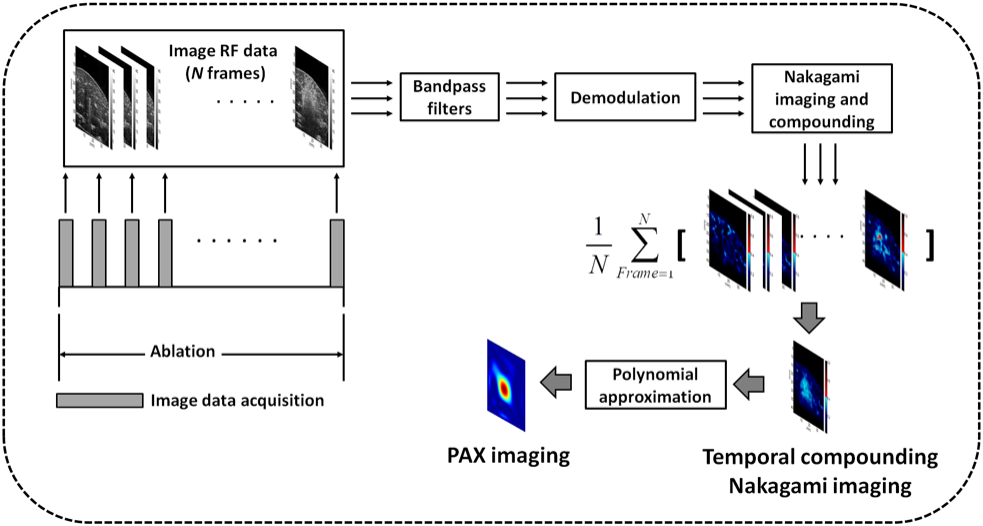
The algorithmic scheme for Nakagami imaging proposed in this study for real-time monitoring RFA. Compared with the conventional Nakagami image, the new algorithm applies frequency and temporal compounding of Nakagami images for an enhanced visualization of RFA-induced ablation zones.

As mentioned in the Introduction, a new-generation Nakagami imaging technique for monitoring RFA necessitates proposing a novel algorithmic scheme that can operate smoothly on a standard ultrasound system to facilitate real-time ablation zone visualization that has improved sensitivity. The newly proposed algorithm illustrated in Fig. 2 involves the following five steps:

- At each time point during RFA, RF image data consisting of numerous scan lines (i.e., backscattered signals) are acquired from the tissue.
- Frequency diversity is conducted by passing the raw image data through two bandpass filters (BPFs), namely BPF I and BPF II, to produce two frames of filtered data (Data I and Data II). The envelope images of Data I and Data II (Envelope I and Envelope II) are obtained from the absolute value of the Hilbert transform of Data I and Data II, respectively.
- For Envelope I and Envelope II, the sliding window technique is applied to obtain their corresponding Nakagami images, *Img*_1_ and *Img*_2_. These two Nakagami images (*Img*_1_ and *Img*_2_) are then averaged to yield a compounding Nakagami image *Img*_*C*_. Steps (ii) and (iii) are the frequency diversity and compounding (FDC) technique, which was proposed to improve the sensitivity of the Nakagami parameter when detecting the variation in scatterer concentration [32]. Therefore, the Nakagami image *Img*_*C*_ can be regarded as the frequency-compounding Nakagami image.
- During RFA, multiple frames of *Img*_*C*_ images can be obtained at various time points. Gas bubble generation in the ablation zone is continual during RFA. To collect sufficient backscattering information from bubbles and improve ablation zone visualization, after RFA is terminated, the *Img*_*C*_ images acquired at various time points are averaged to obtain the temporal compounding Nakagami image $\bar{Img_{C}}$.
- To comprehensively describe the change in image features induced by RFA, polynomial approximation is applied to the $\bar{Img_{C}}$ image to produce a polynomial approximation (PAX) image. The PAX algorithm of a Nakagami image was described in a previous study [26].

### Real-time Nakagami imaging system

Constructing Nakagami imaging requires only beamformed RF signals. Thus, the proposed algorithmic scheme is compatible with the standard pulse—echo ultrasound system. In this study, a commercial clinical ultrasound scanner (Model 3000, Terason, Burlington, MA, USA) was used as a system platform. The reason for using the Terason system was that the manufacturer provides a software development kit (SDK), enabling users to combine algorithms with the system for real-time imaging and online analysis.

**Fig 3:**
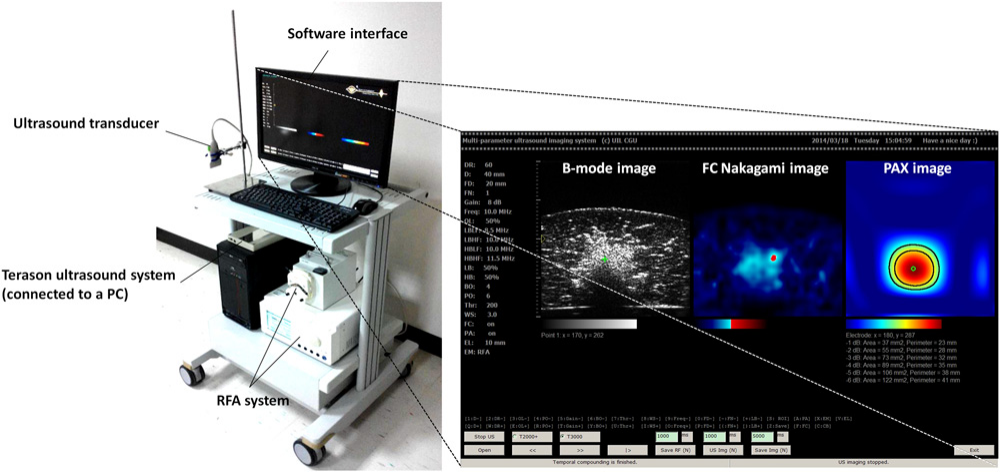
Real-time Nakagami imaging system for monitoring RFA. The proposed algorithm is integrated into a PC-based clinical ultrasound scanner (Terason ultrasound).

The Terason ultrasound imaging system was connected to a personal computer (PC) (CPU: AMD Phenom II X4 945, 3.01 GHz) operating on Windows XP, and we developed the software on the system by using C++ language with OpenCV 2.4.3. OpenCV is an open source computer vision library that has been widely used worldwide (http://opencv.org/). OpenCV was employed because it performs well in image matrix manipulation [33]. When the software is used, image beamformed RF data at a sampling rate of 30 MHz are accessed online from the ultrasound scanner through an IEEE 1394 interface. The RF data are then processed by the computational core for real-time B-mode, frequency compounding Nakagami, and PAX imaging. The frame rate was 1–2 fps when three modes of images were concurrently displayed to monitor RFA. Fig. 3 shows the real-time Nakagami imaging system.

### Experimental validations

To test the performance of the real-time Nakagami imaging system in monitoring RFA and to examine the effects of tissue type on ablation zone visualization, samples of porcine liver and pork tenderloin obtained from a local market were measured. The liver consists of numerous liver cells, and the tenderloin has muscle fibers; these two types of tissues were used because clinical RFA is widely applied to ablations of these types of tissue.

The RFA system (Model VIVA RF generator, Starmed Co. Ltd., Goyang, Gyeonggi, South Korea) used to ablate the tissues in the experiments comprised a cool-tip RF electrode (Model 17–20V15–40, Starmed Co. Ltd.), RF generator, peristaltic pump, cables, and other accessories. The RF output power is adjustable within the range of 0 to 200 W, and the operation frequency is 480 kHz. The active tip length (ATL) of the RF electrode was adjusted to 0.5, 1.0, and 1.5 cm. The real-time Nakagami imaging system was equipped with a 7.5-MHz linear array transducer (Model 10L5, Terason), the pulse length of which was approximately 0.7 mm.

Before the experiments, the tissue sample was cut to an appropriate size and placed in an acrylic case that was filled with a saline solution of 0.9% NaCl. At the bottom of the case, a gel phantom was created to hold the samples and circumvent the influence that the strong reflection echoes returning from the bottom of the case exerted on the ultrasound backscattered signals received from the tissue. To enable the operation of the RFA system, a metal board was attached to the wall of the case as a grounding pad. In the experiments, the RF needle electrode was inserted into the sample through a small hole created in the case wall. To prevent the saline solution from leaking from the hole, clay materials were used to plug the hole. The ultrasound transducer was immersed in the saline solution and placed above the sample. The distance between the transducer and the sample was determined according to the focal length of the transducer, which was adjustable; thus, the sample could be located in the focal zone of the ultrasound for scanning.

The RFA system was subsequently activated in the default automatic mode, which began at 50 W/min and automatically increased by 10 W/min until the first RF pulse paused because of high tissue impedance. The system then generated a sequence of RF pulses as a function of time. The treatment time of the automatic mode was 12 min. During RFA, the ablated sample was monitored using the proposed system in an online regime. For each of the three ATLs (0.5, 1.0, and 1.5 cm), six porcine liver samples (*n* = 18) and two muscle tissues (*n* = 6) were ablated in the experiments.

### Imaging parameters

The dynamic range of B-mode imaging was set at 60 dB. For Nakagami imaging, the window overlap ratio was set as 50%, and the sidelength of the window was 2.1 cm, which was three times the transducer pulse length. The passbands of BPF I and BPF II were 5.5–7.5 MHz and 7.5–9.5 MHz, respectively. To reduce the computational loading, temporal compounding Nakagami imaging $\bar{Img_{C}}$ was created using the *Img*_*C*_ images corresponding to the 1st, 2nd, …, 12th min of the ablation procedure. According to a suggestion in a previous study [26], the order for PAX imaging was set at 7.

### Data analysis and comparison

**Fig 4:**
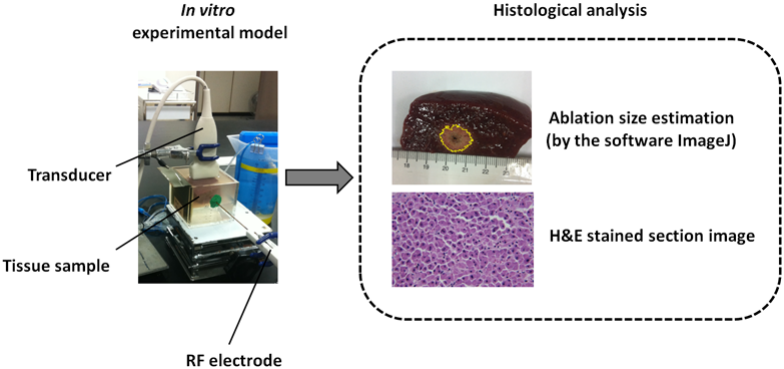
After RFA, the tissue sample was cut along the ultrasound imaging plane for histological observations, and the size of the ablation zone was estimated using ImageJ.

After RFA, the tissue samples were cut along the ultrasound imaging plane and photographed. The photographs were analyzed using ImageJ software (http://imagej.nih.gov/ij/) to measure the sizes of the RFA-induced ablation zones (Fig. 4). To further examine the change in cell morphology caused by RFA, each tissue sample was fixed in 10% neutral buffered formalin, embedded in paraffin, and sliced into 4-μm-thick sections for histological analysis that involved the hematoxylin and eosin (H&E) staining method. An experienced pathologist identified whether tissue necrosis caused by RFA was induced effectively. In addition, the real-time Nakagami imaging system automatically calculated the size of the ablation zone according to the -1, -2, -3, -4, -5, -6 dB contours of the detected ablation zone in the PAX image. The sizes of the ablation zone obtained from tissue section and PAX images were compared to calculate the correlation coefficient.

## Results

**Fig 5:**
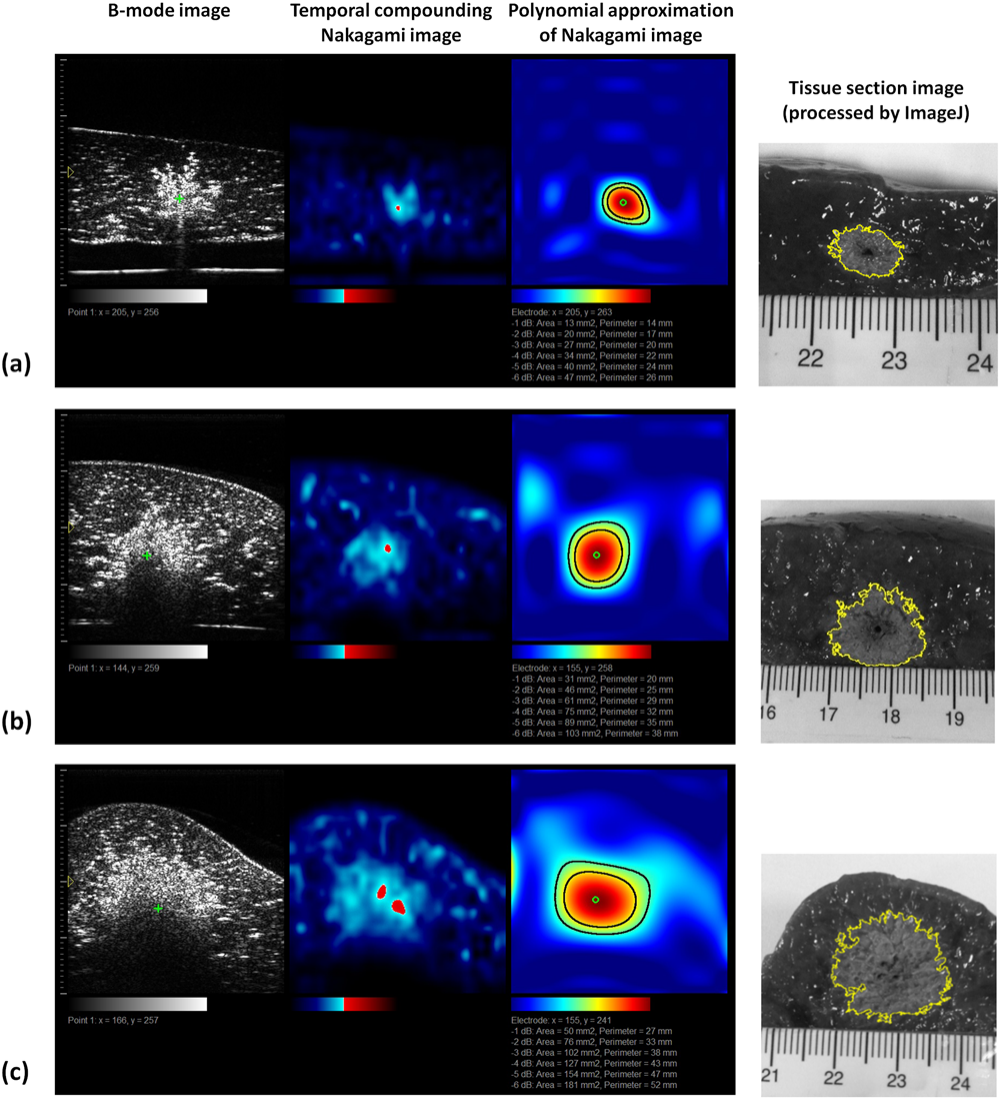
B-mode, temporal compounding Nakagami $\bar{Img_{C}}$, PAX, and tissue section images of RFA-induced ablation zones in porcine liver when the ATL = 0.5 cm (a), 1.0 cm (b), and 1.5 cm (c). The green crosshair in the B-mode images indicates the location of the RF electrode.

**Fig 6:**
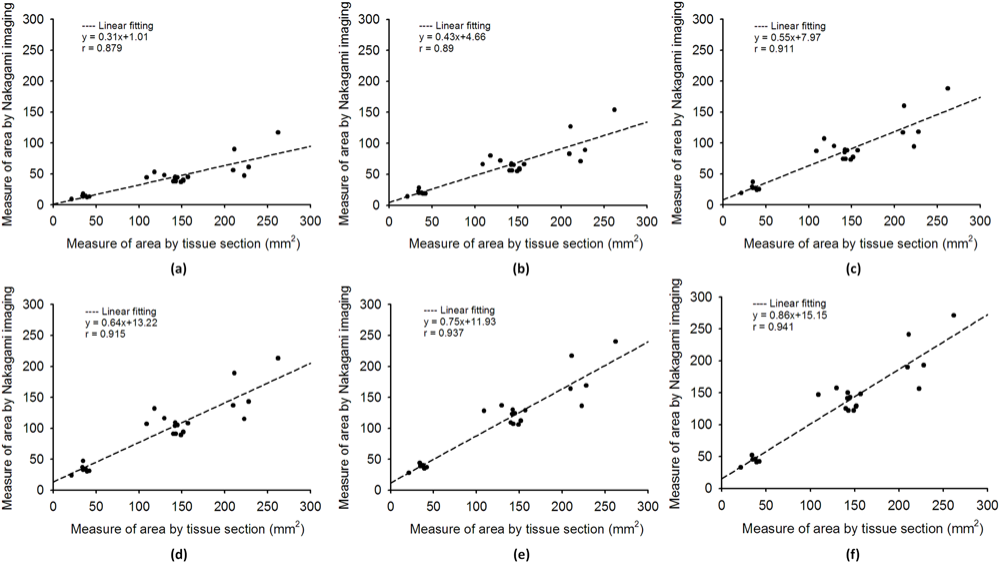
The relationship between the sizes of the ablation zone in the tissue section measured using ImageJ and the (a) –1, (b) –2, (c) –3, (d) –4, (e) –5, and (f) –6 dB contours in the PAX images (*n* = 18). The ablation size estimated using the—6 dB contour in the PAX images had the highest correlation with that measured using the tissue section images (*r* = 0.941).

**Fig 7:**
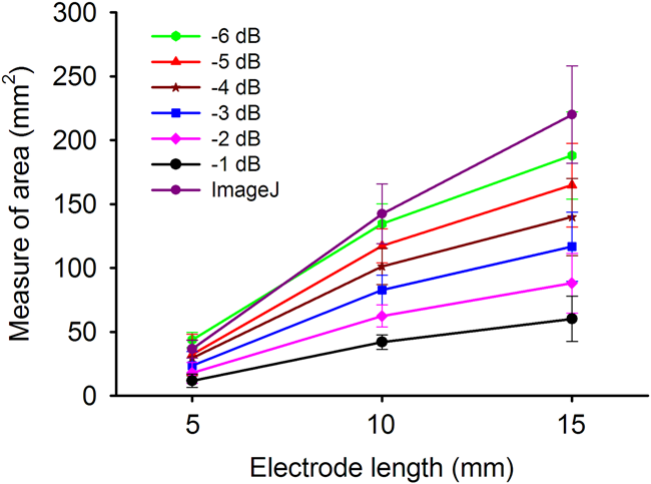
The ablation sizes estimated using the PAX and tissue section images as a function of RF needle length. The ablation size estimated using the—6 dB contour in the PAX image is the nearest to that measured using ImageJ.

**Fig 8:**
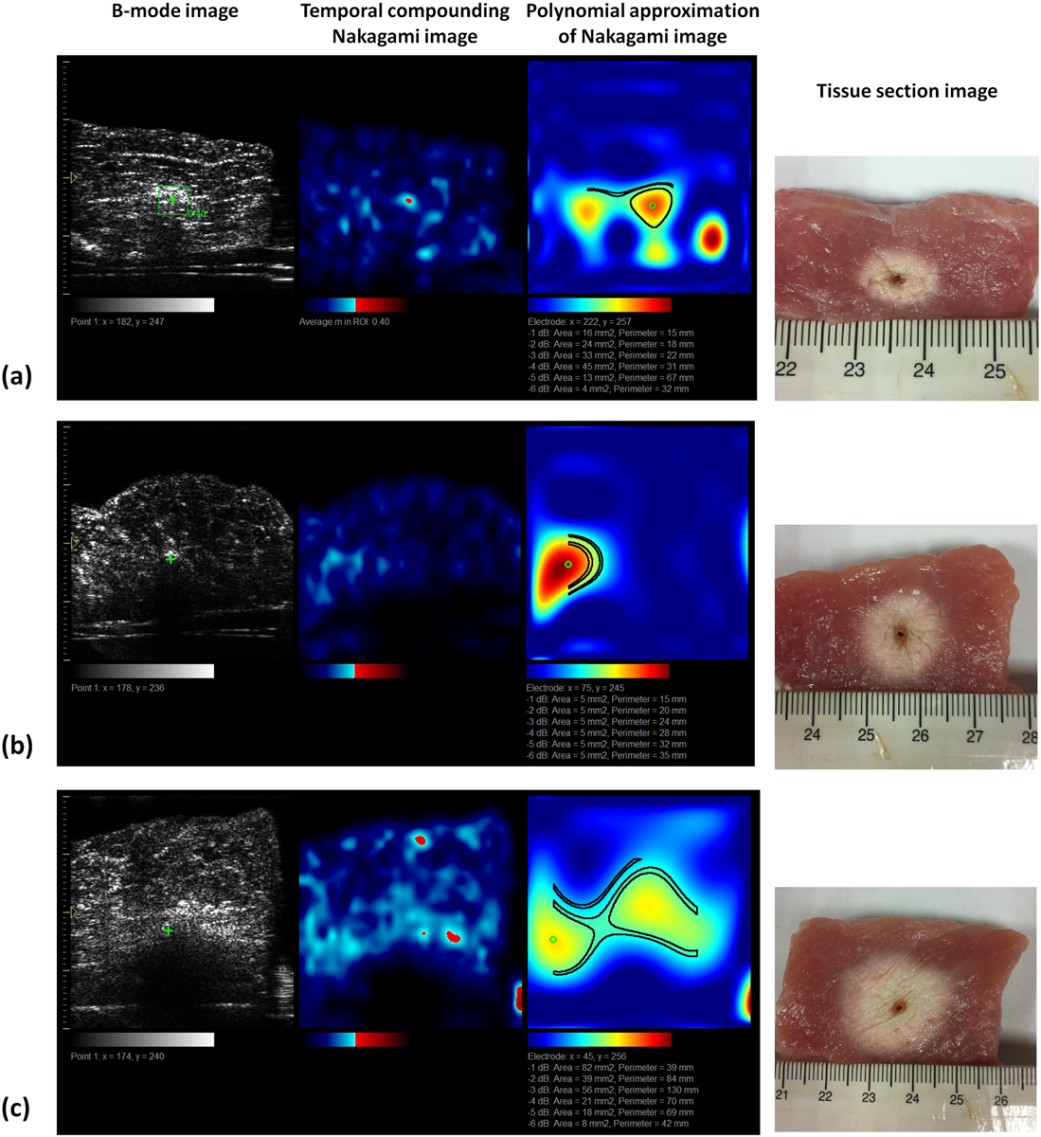
B-mode, $\bar{Img_{C}}$, PAX, and tissue section images of RFA-induced ablation zones in the pork tenderloin when the ATL = 0.5 cm (a), 1.0 cm (b), and 1.5 cm (c). The results indicated that Nakagami imaging cannot monitor the RFA of muscle fiber tissues.

**Fig 9:**
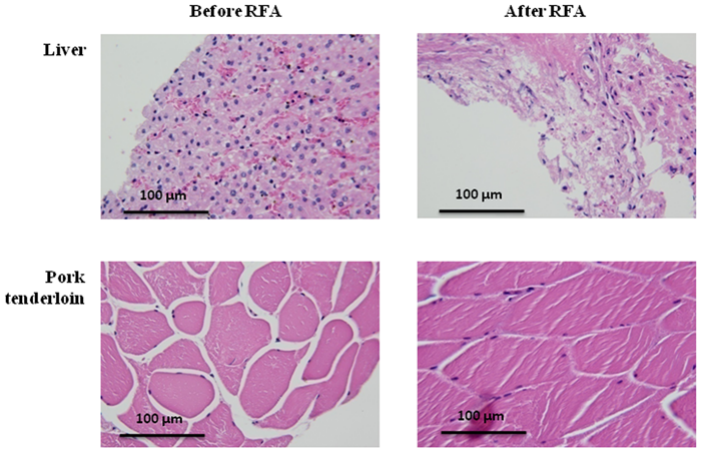
H&E stains of the porcine liver and muscle tissue before and after RFA.

Fig. 5 depicts the B-mode, temporal compounding Nakagami $\bar{Img_{C}}$, PAX, and tissue section images of in vitro RFA-induced ablation zones in porcine liver when the ATL was 0.5, 1.0, and 1.5 cm. The green crosshair in the B-mode image indicates the location of the RF electrode. The yellow contour in the tissue section image was generated using the ImageJ software and indicates the ablative margin. A strong shadowing effect occurred on the RFA-induced lesion, and thus, using the B-mode image to describe the ablation zone was difficult. By contrast, the $\bar{Img_{C}}$ image was markedly less affected by the shadow effect when the ablation zone was characterized. In particular, using the PAX image based on the $\bar{Img_{C}}$ enabled effectively visualizing the change in the backscattered statistics and estimating the ablation zone. Fig. 6 shows the relationship between the sizes of the ablation zone estimated using the tissue section measured in ImageJ and those estimated using the -1, -2, -3, -4, -5, and -6 dB contours of the PAX images (*n* = 18). The ablation size estimated according to the -6 dB contour in the PAX images had the highest correlation with that measured from tissue section images (*r* = 0.941). To confirm this observation, we further examined the ablation sizes estimated using the PAX and tissue section images as a function of RF needle length, as shown in Fig. 7. The ablation size estimated using the—6 dB contour in the PAX image was the nearest to that measured in ImageJ. Fig. 8 shows the B-mode, $\bar{Img_{C}}$, PAX, and tissue section images of RFA-induced ablation zones in the pork tenderloin in vitro when the ATL was 0.5, 1.0, and 1.5 cm. The results indicated that Nakagami imaging was unable to monitor the RFA of muscle fiber tissues. Fig. 9 depicts the H&E stained images before and after RFA of the liver and muscle tissues. Apparently, the cell structures and sizes in the liver and muscle tissues differed. The Discussion section uses Fig. 9 to discuss the reason that the ultrasound Nakagami imaging operated differently when monitoring the liver and muscle tissues.

## Discussion

### Significance of this study

This study is an extension of a previous study [26] that investigated the feasibility of using ultrasound Nakagami imaging to monitor RFA by detecting changes in the backscattered statistics induced by bubbles formed in the ablation zone. Compared with that of the previous study, the significance of this study is the following engineering innovations and scientific contributions.

First, a real-time ultrasound Nakagami imaging system for RFA monitoring was effectively implemented using a commercial ultrasound scanner. In this system, the algorithm of Nakagami imaging differed from that of conventional Nakagami imaging. The proposed algorithm is based on the combination of Nakagami imaging, FDC, and temporal compounding techniques. As mentioned, the FDC was initially proposed for improving the sensitivity of the Nakagami parameter to separate different number densities of scatterers [32,34]. This study is the first to combine the FDC with Nakagami imaging to create FDC-based Nakagami imaging (i.e., *Img*_*C*_ images). In particular, the use of temporal compounding ($\bar{Img_{C}}$ image) enabled the backscattering information from the bubbles induced during RFA to be effectively used for enhanced ablation zone visualization. Because of the software design and the limitations of computational resources in the PC, the interface did not additionally display the conventional Nakagami image.

In addition to this engineering innovation, the effects of tissue type on ultrasound Nakagami imaging when visualizing the RFA-induced ablation zone were examined; this aspect has not been investigated before. The experimental results indicated that ultrasound Nakagami imaging effectively visualized the RFA of the liver tissue, but was not applicable to monitoring fibrotic muscle tissue. This finding is a beneficial reference for future clinical applications of Nakagami imaging to monitor RFA. Possible mechanisms for why the ultrasound Nakagami imaging is unsuitable for monitoring the RFA of muscle tissue are discussed as follows.

### Effect of tissue type

Most of the liver volume is occupied by parenchymal cells, commonly referred to as hepatocytes. A typical hepatocyte forms a cubical cell with 20–30-μm sides. The muscle tissue consists of fibers that are also known as myocytes or muscle cells. Myocytes are long, tubular cells that develop from myoblasts to form muscles through myogenesis. Refer to Fig. 9. Compared with muscle cells, liver cells are much smaller. Because the strength of ultrasound backscattering depends on the size of scatterers, liver cells may be considered weak scatterers exhibiting low echogenicities. Thus, when RFA is applied to liver tissue, the amplitude of the backscattered signals from the thermal-induced bubbles is substantially larger than that from unablated liver cells. This phenomenon facilitates a favorable image contrast during Nakagami imaging that enables visualizing ablation zones. By contrast, muscle fibers are relatively strong scatterers, generating stronger backscattered echoes. Even when thermal-induced bubbles exist in an ablation zone, the dynamic range in the amplitude of signals returned from ablation and unablated zones may be limited. In this condition, the image contrast of ultrasound Nakagami imaging may be insufficient for monitoring ablation zones, and monitoring the RFA of muscle tissues may be ineffective. The other possible reason is that hepatic cells are rich in intercellular fluids compared with muscle fibers. Gas bubbles are more easily generated in liver tissues than in muscle fiber during RFA. Additional gas bubbles generated in ablation zones can create strong echoes that enhance the contrast of Nakagami imaging during RFA.

### Comparison with ultrasound elastography systems

Currently, ultrasound elastography is the most studied approach for monitoring RFA. Mechanical testing measurements of tissues revealed that tissue stiffness increases as the temperature and ablation duration increases [7]. Ultrasound elastography is used to monitor RFA because the RFA-induced ablation zone is stiffer than normal, untreated tissue. However, when heat-induced gas bubbles are generated during the RFA procedure, the bubble-related signals can cause artifacts in ultrasound elastography of ablation zones [15,17,35]. For instance, loss of thermal lesion boundary information on ultrasound elastography images was observed in regions in which attenuation occurred because of bubble effects [35]. Thus, ultrasound elastography techniques have been suggested for monitoring RFA post-ablation or during ablation before substantial bubbles form [15,17].

Unlike that of ultrasound elastography, the working principle of Nakagami imaging in monitoring RFA is based on detecting thermal-induced bubbles by analyzing backscattered statistics. Bubble formation in tissues triggers a change in the statistical distribution of backscattered signals [26]. In other words, when using RFA with a high power to ablate tissues and bubble formation is unavoidable, ultrasound Nakagami imaging may be an alternative solution to effectively monitor ablation zones and evaluate tissue necrosis.

In addition, most ultrasound elastography systems apply specific ultrasound architecture instead of conventional ultrasound platforms (e.g., the shear-wave elastography system that uses ultrafast plane-wave imaging) [36]. The low availability of these systems is a considerable problem in numerous developing countries, particularly in regions with limited resources [24,25]. However, the Nakagami imaging system for RFA monitoring presented in this paper was implemented on a conventional pulse—echo ultrasound system, which may enable Nakagami imaging to be more accessible compared with ultrasound elastography systems.

### Future studies

According to our results and findings, future studies should (i) apply effective strategies to increase the frame rate of real-time Nakagami imaging (e.g., use a graphics processing unit or coding optimization) and (ii) combine the real-time Nakagami imaging system with curve array transducers. However, the central frequency of curve transducers is typically between 3 and 3.5 MHz, which is lower than that of the linear array transducer we applied in this study. Using a low-frequency ultrasound may reduce the sensitivity of the Nakagami parameter in tissue characterization [37]. Thus, determining an additional solution to enhance the parameter sensitivity is necessary. (iii) Future studies should validate the proposed system by using in vivo animal models or clinical trials to consider practical concerns such as the blood perfusion and respiratory motion that are common in clinical RFA procedures.

## Conclusion

This study developed a real-time ultrasound Nakagami imaging system for monitoring RFA. In addition, the effects of tissue type on the performance of using Nakagami imaging to monitor RFA were examined by conducting experiments with liver and muscle tissues in vitro. The engineering innovation and scientific contributions are summarized as follows: (i) A novel algorithmic scheme for Nakagami imaging based on the frequency and temporal compounding techniques was proposed for an enhanced visualization of RFA-induced ablation zones. (ii) The proposed algorithm can operate on a standard clinical ultrasound scanner to monitor RFA in real time for evaluating ablation zones by analyzing changes in backscattered statistics. (iii) The Nakagami imaging system performs well in the ablation zone visualization of liver tissues, but is not applicable to monitoring RFA of muscle fiber tissues. The discrepancy in monitoring RFA of liver and muscle tissues may be due to the difference in both the echogenicity and tissue properties between the two types of tissues. Future studies should focus on increasing frame rate, enhancing parameter sensitivity, and conducting experiments on animal or clinical models.
